# Comparison of MOLLI and ShMOLLI in Terms of T1 Reactivity and the Relationship between T1 Reactivity and Conventional Signs of Response during Adenosine Stress Perfusion CMR

**DOI:** 10.4274/balkanmedj.galenos.2020.2019.12.161

**Published:** 2020-08-11

**Authors:** Esin Gezmiş, Charles Peebles, Andrew Flett, Ausami Abbas, Stephen Harden, James Shambrook

**Affiliations:** 1 Department of Radiology, Başkent University Hospital İzmir Practice and Research Center, İzmir, Turkey; 2 Department of Cardiothoracic Radiology, Southampton University Hospital, Southampton, United Kingdom; 3 Department of Cardiology, Southampton University Hospital, Southampton, United Kingdom

**Keywords:** Adenosine stress adequacy, cardiac magnetic resonance, coronary heart disease, MOLLI, ShMOLLI, T1 mapping

## Abstract

**Background:**

One of the most important techniques of cardiac magnetic resonance in assessment of coronary heart diseases is adenosine stress myocardial first-pass perfusion imaging. Using this imaging method, there should be an adequate response to the drug adenosine to make an accurate evaluation. The conventional signs of drug response are not always observed and are often subjective. Methods based on splenic perfusion might possess limitations as well. Therefore, T1 mapping presents as a novel, quantitative and reliable method. There are several studies analyzing this newly discovered property of different T1 mapping sequences. However most of these studies are enrolling only one of the techniques.

**Aims:**

To compare modified look-locker inversion recovery and shortened modified look-locker inversion recovery sequences in terms of T1 reactivity and to determine the relationship between T1 reactivity and conventional stress adequacy assessment methods in adenosine stress perfusion cardiac magnetic resonance.

**Study Design:**

A cross-sectional study using STARD reporting guideline.

**Methods:**

Thirty-four consecutive patients, who were referred for adenosine stress perfusion cardiac magnetic resonance with suspect of myocardial ischemia, were prospectively enrolled into the study. Four patients were disqualified, and thirty patients were included in the final analysis. Using both modified look-locker inversion recovery and shortened modified look-locker inversion recovery, midventricular short axis slices of T1 maps were acquired at rest and during peak adenosine stress before gadolinium administration. Then, they were divided into six segments according to the 17-segment model proposed by the American Heart Association, and separate measurements were made from each segment. Mean rest and mean stress T1 values of remote, ischemic, and infarcted myocardium were calculated individually per subject. During adenosine administration, patients’ heart rates and blood pressures are measured and recorded every one minute. Adenosine stress perfusion images were examined for the presence of splenic switch-off.

**Results:**

There was a significant difference between rest and stress T1 values of remote myocardium in both modified look-locker inversion recovery and shortened modified look-locker inversion recovery (p<0.001). In both modified look-locker inversion recovery and shortened modified look-locker inversion recovery there was no significant correlation between T1 reactivity and heart rates response (modified look-locker inversion recovery p=0.30, shortened modified look-locker inversion recovery p=0.10), blood pressures response (modified look-locker inversion recovery p=0.062, shortened modified look-locker inversion recovery p=0.078), splenic perfusion (modified look-locker inversion recovery p=0.35, shortened modified look-locker inversion recovery p=0.053). There was no statistically significant difference between modified look-locker inversion recovery and shortened modified look-locker inversion recovery regarding T1 reactivity of remote (p=0.330), ischemic (p=0.068), and infarcted (p=0.116) myocardium.

**Conclusion:**

T1 reactivity is independent of the other stress response signs and modified look-locker inversion recovery and shortened modified look-locker inversion recovery do not differ in terms of T1 reactivity.

Cardiac magnetic resonance (CMR) has proven to be a valuable imaging modality that can be used to both diagnose and differentiate coronary heart diseases (CHD) from other cardiomyopathies. One of the most important techniques of CMR in assessment of CHD is adenosine stress myocardial first-pass perfusion imaging. This type of imaging has high sensitivity (80-90%) and ability to demonstrate ischemic but not infarcted areas which are more likely to be viable (1). Using this imaging method, there should be an adequate response to adenosine to make an accurate evaluation and to avoid false negative findings (2,3). The increase in heart rate (HR) by 10 bpm and the decrease in systolic blood pressure (SBP) by 10 mmHg are signs of hemodynamic response. Meanwhile, the patient can experience flushing, chest tightness, and headache which might also be a proof of adequate adenosine stress (4,5). However, those findings are not always observed and are often subjective. Other recently recommended methods are visual evaluation of splenic perfusion, in which failure of splenic switch-off is an indicator of inadequate pharmacologic stress, and splenic T1 reactivity, which is the difference between rest and stress T1 values of spleen (5,6). However, these might have limitations as well. Therefore, an additional quantitative and reliable method may be beneficial. Recent studies showed that T1 values of normal/remote myocardium increase during adenosine stress compared to rest. Moreover, rest/stress T1 mapping can differentiate between normal/remote myocardium and infarcted or ischemic myocardium without the need for gadolinium contrast (7,8). The modified look-locker inversion recovery (MOLLI) sequence, which was created by Messroghli et al. (9) in 2004, and the faster and more robust shortened modified look-locker inversion recovery (ShMOLLI) sequence, which was proposed by Oxford group in 2010, are two common mapping techniques used for quantification of myocardium T1 values (10). There are several studies analyzing this newly discovered property of different T1 mapping sequences. However, most of these studies are enrolling only one of the techniques. We aimed to compare MOLLI and ShMOLLI in terms of T1 reactivity, and to determine the relationship between T1 reactivity and conventional stress adequacy assessment methods in adenosine stress perfusion CMR.

## MATERIALS AND METHODS

All patients gave a written informed consent to participate in the study and ethical approval was granted by the National Research Ethics Service (13/YH/0223). The study has been performed in accordance with the ethical standards as laid down in the 1964 Declaration of Helsinki and its later amendments or comparable ethical standards. This is a cross-sectional study and STARD reporting guidelines were used (11).

### Patient population

Thirty-four consecutive patients, who were referred for adenosine stress perfusion CMR with suspect of a myocardial ischemia, between December 2016 and February 2017, were prospectively enrolled into the study. Four patients were disqualified based on exclusion criteria, which were general CMR contraindications, mapping motion artifacts, technical failures, underlying cardiomyopathy, and caffeine intake. Thirty patients were included in the analysis.

### Cardiac magnetic resonance imaging protocol

A 1.5 T scanner (Magnetom Avanto; Siemens Healthcare, Germany) was used on all patients. Firstly, standard cine images and STIR sequences were acquired in three cardiac planes (short axis (SA); two chambers; four chambers). Then, using both MOLLI (WIP780B), with 5(3)3 sampling scheme and ShMOLLI, basal, midventricular, apical SA slices of T1 maps were performed at rest (10,12). During administration of adenosine (140 μg/kg/min, intravenously for ≥3 to 6 min), midventricular SA slices of both MOLLI and ShMOLLI during peak adenosine stress were acquired. In order not to prolong scanning time and patients’ exposure to adenosine, which may increase the discomfort and risk of complication, only midventricular SA slices of stress T1 maps were obtained. The mid-ventricular slice was preferred, because more myocardium is shown (six segments), and there was less blood pool contamination and artifact. During each examination, patient’s drug response was assessed by the responsible consultant radiologist. However uptitration of adenosine was not needed in any of the patients based on the findings of symptomatic and hemodynamic responses. Following stress T1 maps, first-pass perfusion imaging was performed, on matching SA slices to rest T1 maps, during peak adenosine stress with an intravenous bolus of gadolinium (0.1 mmol/kg). Late gadolinium enhancement (LGE) imaging was performed 10 min after an additional bolus of gadolinium (0.1 mmol/kg). During adenosine administration, HR and SBP of the patients were measured and recorded every one minute.

### Imaging analysis

**Figure 1:**
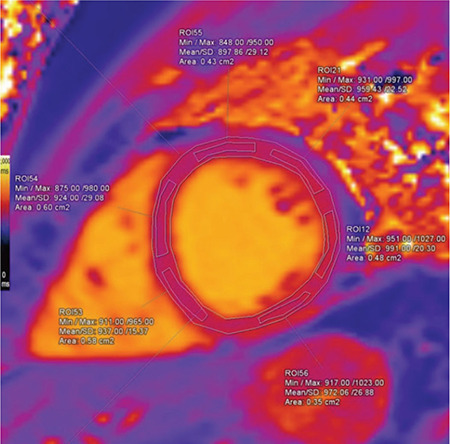
The endocardium and epicardium were manually outlined. Separate measurements were made from each segment.

**Figure 2:**
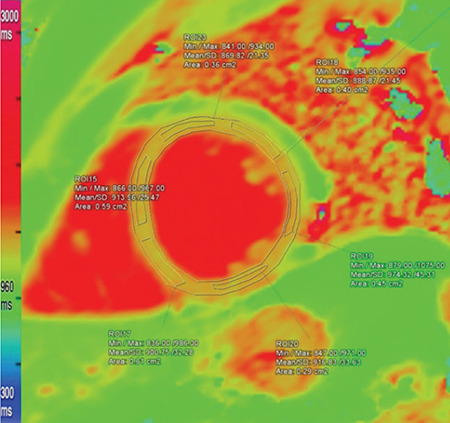
The endocardium and epicardium were manually outlined. Separate measurements were made from each segment.

**Figure 3:**
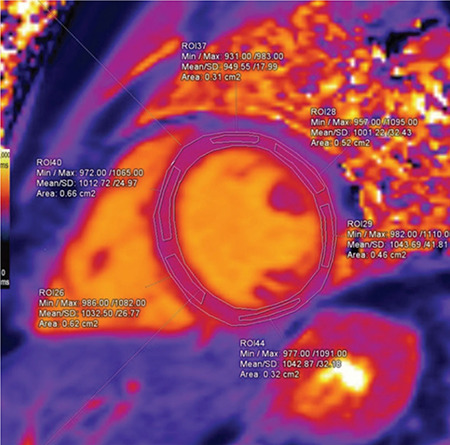
The endocardium and epicardium were manually outlined. Separate measurements were made from each segment.

**Figure 4:**
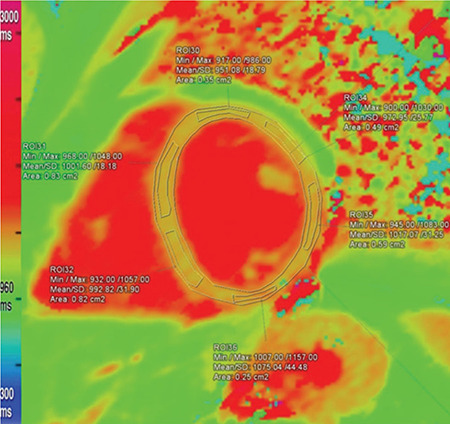
The endocardium and epicardium were manually outlined. Separate measurements were made from each segment.

Using Siemens Syngo.via VA20 software, imaging analysis was performed by two cardiothoracic radiologists: one of them is an accredited consultant and the other is a senior clinical fellow. Both of them were blinded to the patients’ data. The endocardium and epicardium of T1 maps were manually and carefully outlined, avoiding contamination by blood pool and extramyocardial structures. They were divided into six segments according to 17 segment model of left ventricle (LV) proposed by the American Heart Association, and separate measurements were made from each segment (Figure 1, 2, 3, 4) (12). Mean rest and mean stress T1 values of remote, ischemic, and infarcted myocardium were calculated per subject. Segments with reversible perfusion defects, on first-pass stress and rest perfusion images, but without LGE were regarded as ischemic. Segments with subendocardial LGE were defined as infarcted. In ischemic and infarcted segments, regions of interests (ROIs) were placed to affected portions only, excluding remote myocardium found in the same segment. T1 reactivity to adenosine stress was expressed in absolute terms ΔT1(ms)=T1_stress_-T1_rest_ and as percentages ΔT1(%)=ΔT1/T1_rest_x100. T1_rest_ and T1_stress_ represent mean T1 values at _rest_ and during stress, respectively. T1 values of blood pool were also measured in order to exclude partial volume effects. All measurements were firstly made and digitally stored by the senior fellow, and then checked by the consultant. Also, adenosine stress perfusion images were examined for the presence of splenic switch-off, which is a reduction in attenuation of spleen under adenosine stress compered to rest perfusion images (5).

### Statistical analysis

**Figure 5:**
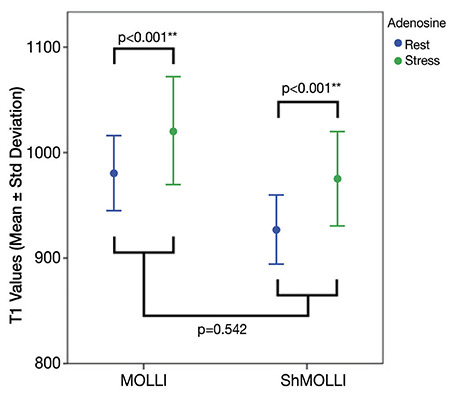
T1 values of remote myocardial segments at rest and stress in MOLLI and ShMOLLI sequences. MOLLI: modified look-locker inversion recovery, ShMOLLI: shortened modified look-locker inversion recovery

All analyzes were performed on SPSS v21. Shapiro-Wilk test was used for the normality test. Comparisons of rest and stress T1 values of remote myocardium were made using multivariate repeated measures ANOVA, where HR response, SBP response, and splenic switch-off were configured as between-subject factors. We calculated ΔT1 of remote, ischemic and infarcted myocardium separately. The resulting data was analyzed using Wilcoxon signed-rank test. Also, with regard to splenic switch-off status, these differences were assessed using Mann-Whitney U test. Correlation between ejection fraction (EF) and remote myocardial ΔT1 was tested using Pearson’s correlation coefficient. Comparison of EF with regard to responses was made using paired t-test. Comparison of MOLLI and ShMOLLI sequences was made using the data from patients, that had rest and stress maps of both MOLLI and ShMOLLI. Comparison of MOLLI and ShMOLLI in terms of ΔT1 was made by using multivariate repeated measures ANOVA for remote myocardium and Wilcoxon signed-rank test for ischemic and infarcted myocardium. Bland-Altman plot was also used for assessment of differences between measurements in remote myocardium (Figure 5). P≤0.05 values were considered statistically significant. Post-hoc power analysis was made based on ΔT1 variable with an effect size of 1.25, an alpha level of 0.05, and a sample size of 26, for MOLLI and ShMOLLI groups. The power is 99%.

## RESULTS

We included 30 patients (23 males and seven females); mean age was 66.07±11.10. Twenty patients had been previously diagnosed with coronary artery disease (CAD). All the patients showed at least one of the conventional response signs. Almost all patients (29/30; 96.7%) experienced symptomatic responses. In 25 (83.3%) patients, hemodynamic response (HR and/or SBP) was detected. In 17 (56.7%) patients, splenic switch-off was seen. Fifteen (50%) patients showed symptomatic response, hemodynamic response (HR and/or SBP), and splenic switch-off. CMR scan results of five patients were normal. Fourteen patients, with previously diagnosed CAD, had infarcted segments, and out of these fourteen patients, eight patients had affected midventricular segments on CMR. Eight patients, with previously diagnosed CAD, had ischemic myocardial segments, and five out of these eight patients, had affected midventricular segments on CMR. Patients’ demographics and characteristics are summarized in Tables 1 and 2.

Midventricular SA slices of MOLLI and ShMOLLI in four and three patients, respectively, could not be achieved because of technical failures. In total, 106 midventricular SA slices of T1 maps were acquired (26 MOLLI sequences and 27 ShMOLLI sequences acquired at rest; 26 MOLLI sequences and 27 ShMOLLI sequences acquired under stress) and subsequently divided into 636 segments. Twenty-three segments (3.62%) (seven MOLLI sequences acquired at rest; 8 MOLLI sequences acquired under stress; two ShMOLLI sequences acquired at rest; six ShMOLLI sequences acquired under stress) were excluded due to off-resonance artifacts, partial volume effects, poor T1 fit on the R2 maps, patient movements, or low signal-to-noise. Six hundred and thirteen segments were included in the final analysis. Eight (26.7%) patients had 12 infarcted myocardial segments in total (In four of the 12 infarcted segments, LGE thickness <50%, whereas the rest (eight segments) showed >50% transmurality). Five (16.7%) patients had six ischemic myocardial segments in total.

**Figure 6:**
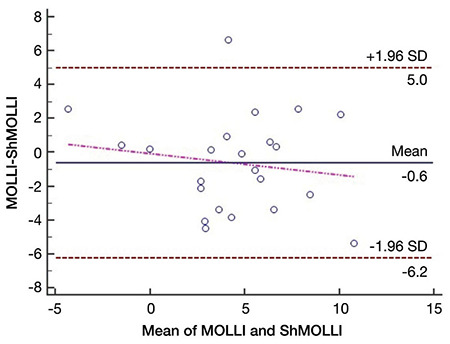
Bland-Altman plot for T1 values of remote myocardium.

When we compared rest and stress T1 values of remote myocardium, there was a significant difference between them in both MOLLI and ShMOLLI (Table 3, Figure 5, 6). There was no significant difference between these sequences with regard to ΔT1 of remote myocardium (p=0.33) (Table 5). When we compared remote and infarcted myocardium in terms of ΔT1, we found significant differences between them in both MOLLI (p=0.017) and ShMOLLI (p=0.043) (Table 4). However, as we divide the infarcted myocardium into two subgroups based on the thickness, we detected that ΔT1 of <50% thick infarcted myocardium showed no significant difference compared to remote myocardium in ShMOLLI (p=0.84) (Table 4). When we compared remote and ischaemic myocardium in terms of ΔT1, we found no significant differences between them in both MOLLI (p=0.14) and ShMOLLI (p=0.35) (Table 4). There was no statistically significant difference between MOLLI and ShMOLLI with regard to ΔT1 in ischaemic and infarcted myocardium (Table 5).

We compared ΔT1 of remote myocardium with HR response, SBP response, and splenic switch-off. In MOLLI, there was no significant correlation between ΔT1 and HR response (p=0.30), SBP response (p=0.062), and splenic switch-off (p=0.35). Similarly, in ShMOLLI, there was no significant correlation between ΔT1 and HR response (p=0.10), SBP response (p=0.078), and splenic switch-off (p=0.053). There were no statistically significant differences between patients with and without splenic switch-off in terms of ΔT1 of remote, ischemic, infarcted myocardium (Table 6). MOLLI and ShMOLLI showed no statistically significant difference in terms of ΔT1 between patients with and without splenic switch-off (Table 7).

There was no significant correlation between ejection fraction (EF) and ΔT1 of remote myocardium in MOLLI (r=0.335; p=0.095), while there was a moderate positive correlation in ShMOLLI (r=0.432; p=0.024). When EF was compared in patients with and without HR response, we found no significant difference (p=0.22). We also found no significant difference in terms of EF between patients with and without SBP response (p=0.16). On the other hand, patients with splenic switch-off were found to have significantly higher EF values in comparison to patients without splenic switch-off (p=0.020) (Table 8).

## DISCUSSION

Mishra et al. (13) proved that HR and SBP correlate poorly with adenosine-induced myocardial hyperemia, and cannot be used to assess the adequacy of stress testing. Splenic markers possess limitations, such as receptor variability, patients having undergone splenectomy, and spleen not being visible in perfusion images. Furthermore we actually do not know how well splenic perfusion correlates with the effects of adenosine on myocardium, which is the main region of interest.

There are many recent studies showing the role of T1 mapping in CMR. One of the main factors that T1 relaxation time depends on is the water content of the tissues, which is highly affected by blood volume (9,10,13,14,15). Hence, coronary vasodilatation, which increases myocardial blood volume (MBV), is expected to prolong T1 and allows detection of microvascular and MBV changes during ischemia (16,17,18,19). Mahmod et al. (20) demonstrated the ability of stress/rest T1 mapping to detect increases in MBV from coronary vasodilatation in patients with severe aortic stenosis and nonobstructive coronary arteries, with complete reversal and normalization after aortic valve replacement. Liu et al. (7) showed that T1 values of normal/remote myocardium increase during adenosine stress compared to rest and also that rest/stress T1 mapping can differentiate between normal/remote myocardium and infarcted or ischemic myocardium. Another research consisting of rest/stress T1 mapping, which is performed by Kuijpers et al. (8), was about the effect of caffeine intake on myocardial perfusion during adenosine stress CMR. They compared between patients who drank coffee and those who did not in terms of ΔT1. Not only did they prove that caffeine inverts the effects of adenosine on myocardium, but also they stated that ΔT1 can be used as a criterion for the validity of the stress induction by adenosine in cardiac MR perfusion studies.

In our study we found that T1 mapping values of remote myocardium increase during stress adequacy indicate coronary hyperemia in both MOLLI and ShMOLLI, regardless of whether there are markers of symptomatic response, hemodynamic response, and splenic switch-off. We found that T1 reactivity in patients with splenic switch-off was not different from T1 reactivity in patients without splenic switch-off (Tables 6,7). Although the number of patients experiencing symptomatic response (29/96.7%) was higher than patients demonstrating T1 reactivity (MOLLI and/or ShMOLLI 28/93.3%), suggesting that it might be a better method, we should keep in mind that symptomatic response is subjective and mainly based on the patients’ expressions (Table 2). We had only one patient (a 76 year-old male, with no history of CAD or diabetes mellitus), who showed solely symptomatic response. However, he was one of the patients who had severe LV impairment, and the adenosine dose could not be increased due to the patient’s discomfort, which was questionable (the probability of inadequate stress was remarked in his CMR report). We observed that T1 values of two patients with severe LV impairment (EF: 22% and 29%) showed no elevation during stress, although, based on conventional methods, both seemed to have adequate stress, with both experiencing signs of symptomatic response and one showing splenic switch-off as well. Although there was not significant correlation between ΔT1 and EF, and there might be other underlying pathology causing blunted T1, one should be cautious while interpreting stress perfusion CMR, keeping in mind that there might not be myocardial hyperemia during adenosine stress. These findings also highlight the suggestion that T1 reactivity could be a better choice to analyze the effect of adenosine on myocardium. To the best of our knowledge the relationship between EF and response to adenosine or ΔT1 has not been investigated briefly and needs to be researched in larger studies.

Our findings confirmed the results of previous studies that state that differentiating between infarcted and remote myocardium can be done using ΔT1 (7,8). Meanwhile, no statistically significant difference was detected between ischemic and remote myocardium in terms of ΔT1, which contradicts the previous studies. The number of ischemic segments was low in our study. Moreover, the thicknesses of all these perfusion deficits were <50%. Although ROIs were carefully placed, we suspect that the interference from remote myocardium found in the same segments might not have been overcome. Additionally when we look at the percentage values of ΔT1, especially in MOLLI, we could detect a slight difference (Table 4), which might have become statistically significant should there have been more and thicker ischemic segments. The specificity and sensitivity of ΔT1 in differentiating segments with <50% thick perfusion deficits from remote myocardial segments, should be investigated in larger studies.

In literature, there are a few studies comparing T1 mapping sequences. However all of them are about native T1 mapping, and most of them were based on phantoms or healthy volunteers. Roujol et al. (21) compared the accuracy, precision, and reproducibility of four T1 mapping sequences (MOLLI, ShMOLLI, SASHA, and SAPPHIRE) by using both a phantom and seven healthy volunteers and reported that the precision of MOLLI was higher than that of ShMOLLI, whereas their reproducibility was similar. Another study by Child et al. (22), which was about the bioequivalence of MOLLI, ShMOLLI, and SASHA in myocardial characterization of diffuse myocardial fibrosis, proved that they differ in their bioequivalence for discrimination between health and disease as well as associations with diffuse myocardial fibrosis. Teixeira et al. (23) compared MOLLI, ShMOLLI, and SASHA in terms of native T1 mapping at 3T and concluded that MOLLI had the smallest overall variability, while SASHA had the best accuracy. Piechnik et al. (10) studied the difference between rest T1 values of MOLLI and ShMOLLI using both phantom and in vivo materials. They revealed that ShMOLLI rest T1s were shorter than MOLLI T1s by 10±16 ms at 1.5 T, and ShMOLLI showed a 15% larger T1 difference between infarcted and unaffected myocardium. Similar to Piechnik et al.’s (10) findings, we observed that both rest and stress ShMOLLI T1s were shorter than MOLLI T1s, except for rest and stress ShMOLLI T1s of <50% thick infarcted myocardium. Moreover, the percentage values of ΔT1 in ShMOLLI were higher in all kinds of myocardial tissues, except in cases of >50% thick infarcted myocardium. However, the difference between rest T1 values of infarcted and remote myocardium was larger in MOLLI compared to ShMOLLI (MOLLI 5.32% and ShMOLLI 4.93%) (Table 5). We did not see statistically significant difference between T1 reactivity of <50% thick infarcted myocardium and remote myocardium in ShMOLLI (p=0.84), whereas, in MOLLI, the difference was statistically significant (p=0.028) (Table 4). Additionally, we noticed that the distinction between ΔT1 values of affected myocardium and ΔT1 values of remote myocardium was more evident in MOLLI compared to ShMOLLI (Table 5). Even though there was no statistically significant difference between MOLLI and ShMOLLI and the obtained result of <50% thick infarct in ShMOLLI might have been due to the intertwined remote myocardium, if researched and confirmed in larger studies, ΔT1 MOLLI might prove to be better in distinguishing the affected tissues from remote ones. Because of the artifacts, we had to exclude 23 segments, of which only eight were of ShMOLLI sequence. This is also in agreement with the published data claiming that ShMOLLI has a smaller noise penalty than MOLLI (10).

Our study population and the number patients with ischemic and/or infarcted myocardium were small. We did not have a control group consisting of healthy volunteers to assess the normal T1 reactivity.

In conclusion, T1 reactivity is independent of systemic, hemodynamic, and splenic switch-off responses. ΔT1 can be used as an additional tool to estimate stress adequacy, as it is robust, reproducible, and introduces objective findings specific to the myocardium. MOLLI and ShMOLLI mapping sequences do not differ in terms of T1 reactivity.

## Figures and Tables

**Table 1:**
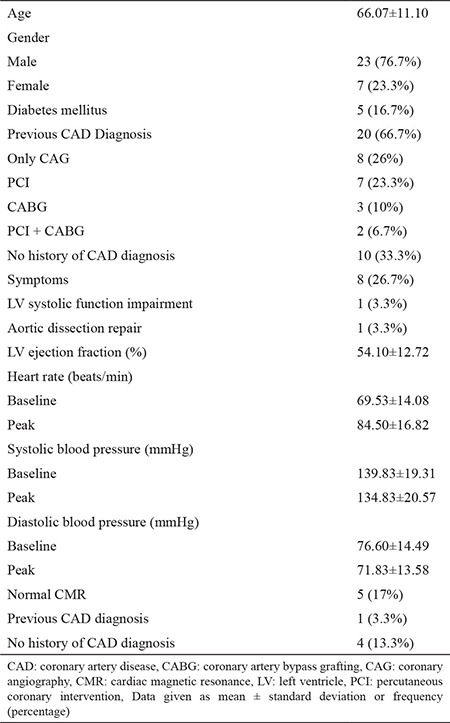
Demographics and characteristics of patients

**Table 2:**
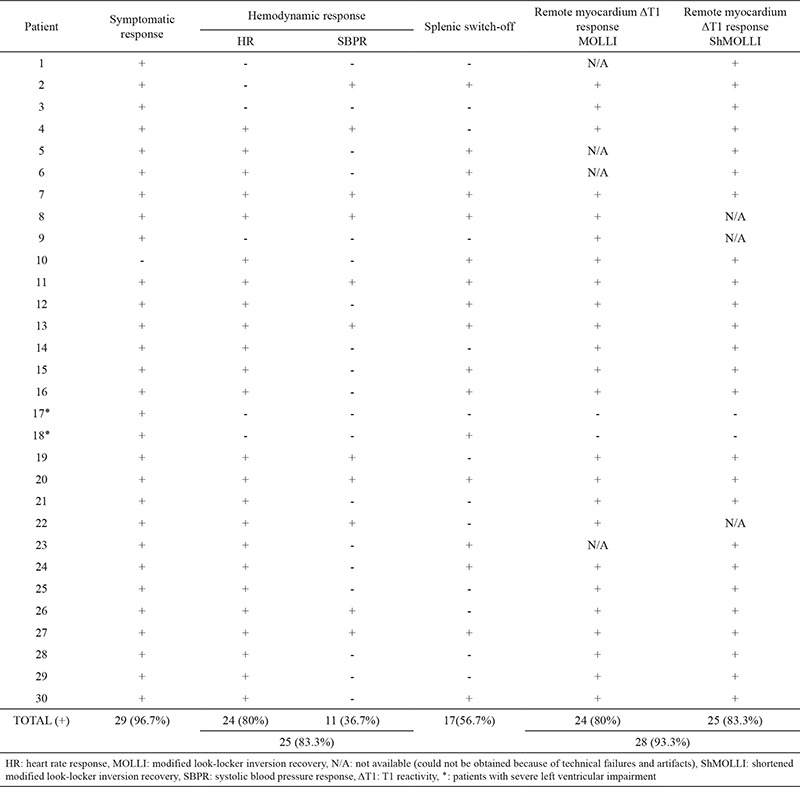
Adenosine drug responses of patients

**Table 3:**
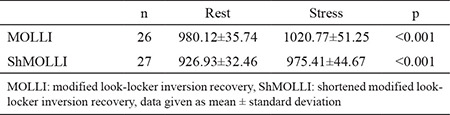
Comparison of T1 values of remote myocardium

**Table 4:**
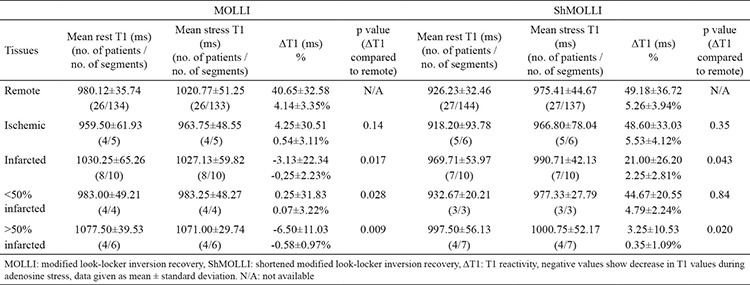
Comparison of T1 values of remote, ischaemic and infarcted myocardium in modified look-locker inversion recovery and shortened modified look-locker inversion recovery

**Table 5:**
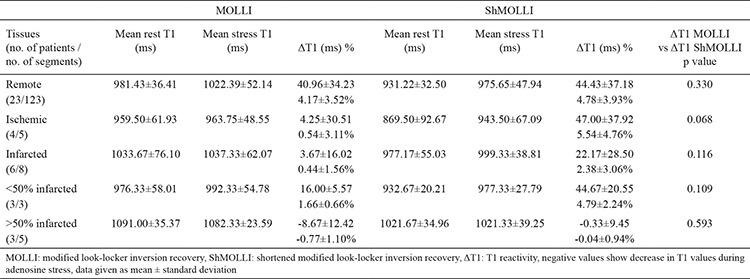
Comparison of modified look-locker inversion recovery and shortened modified look-locker inversion recovery sequences in terms of T1 reactivity of remote, ischaemic and infarcted myocardium in patients with complete sets of rest and stress maps of both MOLLI and ShMOLLI

**Table 6:**
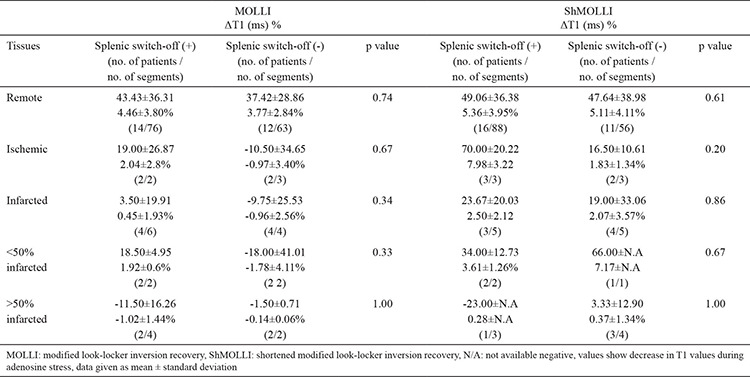
Comparison of ΔT1 of remote, ischemic, and infarcted myocardium with splenic switch-off in MOLLI and ShMOLLI

**Table 7:**
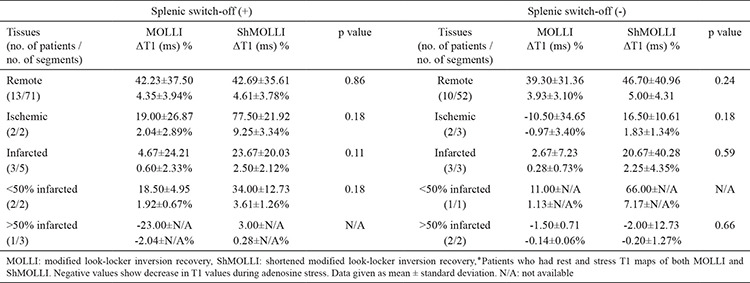
Comparison of MOLLI and ShMOLLI in terms of ΔT1 of remote, ischemic, and infarcted myocardium in patients with and without splenic switch-of٭

**Table 8:**
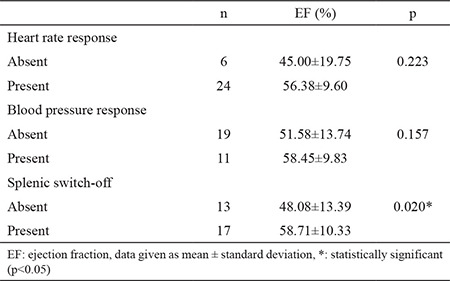
Comparison of ejection fraction with regard to responses
